# Providing Compassionate Care: A Qualitative Study of Compassion Fatigue Among Midwives and Gynecologists

**DOI:** 10.3390/healthcare13222908

**Published:** 2025-11-14

**Authors:** Sarah Vandekerkhof, Laura Malisse, Stefanie Steegen, Florence D’haenens, Hanne Kindermans, Sarah Van Haeken

**Affiliations:** 1 Research and Expertise, Resilient People, University of Applied Sciences Leuven-Limburg, 3590 Diepenbeek, Belgium; laura.malisse@kuleuven.be (L.M.); sarah.vanhaeken@ucll.be (S.V.H.); 2 Faculty of Midwifery, University of Applied Sciences Leuven-Limburg, 3600 Genk, Belgium; stefanie.steegen@ucll.be; 3 Research Group Healthcare and Ethics, Faculty of Medicine and Life Sciences, Hasselt University, 3590 Diepenbeek, Belgium; florence.dhaenens@uhasselt.be (F.D.); hanne.kindermans@uhasselt.be (H.K.)

**Keywords:** compassion fatigue, obstetrics, psychological safety

## Abstract

**Background**: Compassion fatigue (CF) is a state of emotional and physical exhaustion in the caregiving relationship, which can negatively impact patient safety and quality of care. Maternity care professionals are particularly vulnerable to CF due to their continuous empathetic engagement with patients in an unpredictable, high-stress work environment. Despite its significance, research on CF in maternity care is limited. The aim of this study is to explore experiences of CF among maternity care professionals. **Methods**: A thematic analysis of semi-structured in-depth interviews was conducted. The sample consisted of seven midwives and three gynecologists from different hospitals and outpatient care in Flanders (Belgium). **Results**: Experiences, risk factors and protective factors were identified as three organizing themes and further refined into 12 subthemes. Participants showed limited familiarity with the term CF but recognized its symptoms, including emotional exhaustion, reduced empathy, and a diminished ability to provide care, ‘as one normally would’. Key risk factors included high workload, emotional strain from ‘energy-consuming’ patients, fear of errors, and administrative burden. A supportive team environment, compassion satisfaction (CS), job autonomy and personal coping skills were identified as protective factors. Participants emphasized the need to recognize and address signals of CF. **Conclusions**: CF among maternity care professionals is underrecognized but appears to impact both caregiver well-being and patient care quality. Interventions should target awareness, team communication, psychological safety, and organizational context. A multilevel approach—combining individual, team, and systemic strategies—is needed to sustainably mitigate CF in maternity care.

## 1. Introduction

Compassion Fatigue is a state of emotional and physical exhaustion and a loss of coping in caregiving relationships [1,2,3,4,5,6]. It is distinct from secondary traumatic stress (STS), burnout and vicarious traumatization, although these terms are often used interchangeably to describe stress-related complaints among healthcare professionals [2,7,8,9]. Compassion fatigue develops gradually in exposure to patient suffering and the emotional demands of care, rather than from a single traumatic event as in STS [2,7,8,9,10,11]. Unlike burnout, CF arises from the caregiving relationship [1].

Antecedents of CF include chronic exposure to the suffering of others, an inability to maintain professional boundaries, high occupational use of self, high stress exposure and a lack of self-care measures [4]. In healthcare, CF has been studied among nurses, oncologists, and emergency personnel, revealing high prevalence rates and strong associations with burnout, reduced compassion satisfaction and turnover intentions [5,6,7,8,9,10,11]. Although research among maternity care providers is limited, evidence suggests that maternity care professionals are particularly vulnerable to CF due to the unpredictable and emotionally challenging work environment, frequent exposure to traumatic perinatal events and moral distress when witnessing suffering or loss [4,12,13,14,15]. For instance, 75% of midwives in a Greek study were found to be at high risk of CF [16]. Moderate levels of CF have also been observed among gynecologists, although research is scarce [17,18].

Consequences of CF include increased work errors, values questioning, a desire to quit the profession and reduced self-efficacy [4]. This can negatively impact patient safety and quality of care [4]. Patient experiences reflect these issues, with 10% of women reporting negative childbirth experiences characterized by powerlessness and lack of empathetic care [19], which can lead to low self-esteem, breastfeeding difficulties, anxiety and depression and disruptions in mother-child and partner relationships [20,21,22]. To improve care experiences for both caregivers and patients, a deeper understanding of CF is needed. Despite growing recognition of CF as a significant occupational hazard in healthcare, little is known about how maternity care professionals understand and experience this phenomenon in their daily work. The existing literature mainly reports prevalence rates but rarely explores the subjective and contextual dimensions of CF. Therefore, this study aims to explore the experiences of CF among maternity care professionals in Flanders, contributing to a deeper understanding of how CF manifests and affects caregiving relationships in this specific context.

## 2. Materials and Methods

### 2.1. Design

An exploratory qualitative research design was adopted, using semi-structured interviews. Reflexive thematic analysis by Braun and Clarke [23] was selected for its flexibility and atheoretical approach, allowing for an in-depth and nuanced exploration of participants’ experiences with CF within their work contexts. This study adopted a social constructivist and interpretivist epistemology. We assume that knowledge and meaning are co-constructed between researchers and participants and that participants’ stories reflect experiences constructed in social and organizational contexts. The research team consists of clinical psychologists and a midwife-sexologist with training in systemic and solution-focused therapeutic frameworks. Consequently, the researchers approached the data with sensitivity to interactional dynamics, language and the co-construction of meaning in caregiving relationships.

We used several strategies to enhance trustworthiness: investigator triangulation (multiple researchers coded and discussed interpretations), maintenance of an audit trail (field notes, transcripts, coding iterations and meeting reports), regular team discussions and transparent reporting using the consolidated criteria for reporting qualitative research (COREQ) guidelines [24].

### 2.2. Participants and Procedure

This study was conducted in Flanders and included midwives and gynecologists working in gynecological, obstetric and maternity care, encompassing both hospital or primary care settings. Prior experience of CF was not required for participation. A three-tier recruitment was employed: (1) distribution of a research flyer through social media channels, (2) distribution within professional associations, and (3) dissemination through an online survey on CF. In total, 10 professionals agreed to participate, 8 did not respond and 3 interviews were eventually canceled.

Ethical approval was obtained from the Committee for Medical Ethics and the Ethics Committee of Hospital Oost-Limburg (ZOL) in December 2022 (Ref. nr. VT2022-16). Participants received written and oral study information before enrolment. Written informed consent, including explicit permission to video-record interviews and to use anonymized quotes, was obtained from each participant. Video-recordings were deleted after transcription and identifying information was removed from transcripts prior to analysis. Participants could withdraw at any time without consequence. All procedures complied with institutional data-protection requirements.

### 2.3. Data Collection

A semi-structured interview guide was developed including an introduction to establish a relationship, information about the researchers and the research goal and open-ended questions about following topics: (1) personal experiences with CF, (2) individual coping strategies for dealing with CF, (3) work-related factors that are experienced to protect against CF (4) work-related risk factors for the development of CF, (5) the impact of CF on the team, (6) the impact of CF on the personal life, and (7) ideas on what kind of intervention would be helpful to prevent CF. After introduction and informed consent, interviewees were asked about their job content and responsibilities. Next, interviewees were asked to explain what they understood by the concept of CF. After that, the interviewer provided each participant with a formal definition of CF to support deeper reflection. Participants were not given a definition prior to enrolment to avoid priming effects. The interview guide was inspired by previous research on CF risk and protective factors by Harling et al. [25]. The guide was refined after a pilot interview and during data collection to ensure appropriateness for the target group (see Supplementary Material File S1). Authors LM (MSc) and SS (MSc) conducted the interviews. Both interviewers were female researchers trained in interviewing techniques and qualitative research. Interviewers contacted participants via e-mail or phone to provide study information. Following agreement and written informed consent, each participant was interviewed once, individually via Microsoft Teams. Data was collected between January and April 2023. Interviews were visually recorded and field notes were taken. The duration ranged from 52 to 60 min (M = 57).

### 2.4. Data Analysis

Thematic analysis, as outlined by Braun and Clarke [23], guided the analysis of the interviews. A reflexive and primarily inductive approach was used. In line with this interpretative framework, no intercoder reliability was calculated, as coding and theme development were treated as reflexive and the result of meaning-making processes, rather than categorization. Instead, credibility was enhanced through regular discussion and iterative engagement with the data. While coding and theme-generation were data-driven, we acknowledge that the semi-structured nature of the interviews may have shaped the analysis. All versions of the codebook, coding notes and meeting reports were retained as an audit trail. Data collection and analysis proceeded iteratively until thematic saturation was reached, meaning until two consecutive interviews produced no new codes or candidate themes. During the first phase (familiarization), interviews were transcribed verbatim, read, and re-read to facilitate data familiarization. Interview transcripts were not returned to participants for review or correction. Case summary sheets were made (LM and SS) and discussed (LM, SS, SV and SVH) based on transcripts and field notes. In the second phase (initial coding), authors LM, SV, SVH, and SS, independently prepared the initial coding on half of the interviews, using Microsoft Word and Excel. No additional coding software was used. In the third phase (searching for themes), these initial codes were compared and collated into potential themes and subthemes through collaborative discussion among the coders (LM, SS, SV and SVH), resulting in a preliminary codebook. In the fourth phase (reviewing themes), this preliminary codebook was applied to the remaining half of the interviews, with ongoing adjustments and additions to themes as necessary. In the fifth phase (defining and naming themes), the research team collaboratively reviewed and refined each theme and subtheme, resulting in the final thematic framework. In the final phase (report) subthemes were elaborated and illustrated with citations. Quotes in the results section were translated from Dutch to English, with only participant numbers used for identification.

## 3. Results

### 3.1. Participants

**Figure 1:**
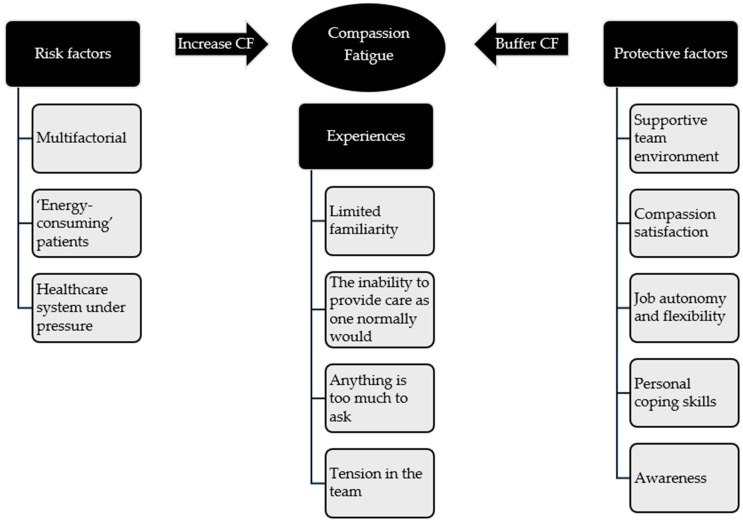
Thematic map of Compassion Fatigue (CF) showing the three major themes (Risk factors, Experiences and Protective factors) with their subthemes. Arrows indicate relations as reported by participants. Note that this map does not imply causal interference.

In total, seven midwives and three gynecologists participated in online interviews. The majority of the sample was female (N = 9), with ages varying from 27 to 59 years (M = 39.3, SD = 8.86). The sample was diverse regarding areas of practice and showed a variation from six to 30 years of experience (M = 14.8, SD = 7.89) (Table 1). Six participants combined their clinical role with positions in education or research. Thematic analysis resulted in three major themes: Experiences, Risk factors and Protective factors. These themes were further refined into subthemes (Figure 1).

### 3.2. Experiences

#### 3.2.1. Limited Familiarity

Initially, professionals were unfamiliar with the term CF. A majority indicated they had never encountered the term before: “I had never heard of it before. […] The first thing that comes to mind is more like burnout” (P1). Participants were more acquainted with the term burnout, which was often confused with CF. Nevertheless, when offered a definition of CF, everyone recognized the issue. Some participants shared personal experiences of CF, while most acknowledged its presence among their teams and colleagues.

#### 3.2.2. The ‘Inability to Provide Care as One Normally Would’

Participants defined experiences of CF as the ‘inability to provide care as one normally would’: “The biggest complaint from midwives was not how busy it was, but that they cannot provide the care they want to provide.” (P9). Respondents note challenges in expressing empathy, which would normally occur naturally, resulting in more routine and autopilot responses and care. This is experienced in patient interactions that are briefer, more agitated and less attuned to patients’ needs. Also, patient demands are considered more excessive when already feeling overwhelmed. Not being able to provide care as one normally would, makes interviewees feel less satisfied about their job. Midwives and gynecologists agree that when there is a noticeable impact on care, it serves as an alarm bell that intervention is needed: “You feel like you really have to put in the effort to be the same caregiver that you usually are” (P8).

#### 3.2.3. Anything Is Too Much to Ask

On an individual level, a decline in patience becomes particularly apparent according to participants. They report feeling overburdened and more susceptible to emotional reactions or irritability than normally: “The bell rings and you go to help that lady and then the bell rings again and again. Normally, I’d have a lot of patience, but after a while, I was like ‘Ah, there’s the bell again’ […] that’s something I don’t recognize in myself because I’m not like that at all.” (P6). Some participants experiencing CF also express challenges in letting go or putting things into perspective.

#### 3.2.4. Tension in the Team

The impact of CF on the team is notable when a team member experiencing CF is frequently absent or takes on fewer tasks than usual. Consequently, other team members must take up additional tasks, increasing their workload. This can lead to irritation, collective rumination, and exhaustion, sending the team atmosphere into a negative spiral: “They’ll often talk about it with colleagues, you know. And then I sometimes think [as a manager], maybe I should try to break that cycle too. Because if one person says ‘I’m not doing well, and this and that’, as a colleague, you’ve got to help pull them out of it, and that’s really exhausting, isn’t it?” (P2).

### 3.3. Risk Factors

#### 3.3.1. Multifactorial

According to interviewees, the onset of CF is multifaceted, resulting from an imbalance between risk factors and available resources on a personal and professional level. Participants linked personal challenges with one’s performance at work: “I think that if your situation at home is also difficult, that can also make you walk more on eggshells at work.” (P8). Consequently, participants believe that maintaining a healthy work–life balance plays an important role in the development of CF.

#### 3.3.2. ‘Energy-Consuming’ Patients

Interviewees point out various patient characteristics which can contribute to the development of CF. Patients who are perceived as overly dependent, anxious or uncooperative are perceived as energy-consuming. A language barrier is also experienced as a challenge. The main challenge encountered by caring for these patients is establishing a meaningful connection: “I think that patients who are very anxious take the most out of me […] If there’s no way to get through them, whether it’s because of a language barrier, a different way of communicating, or simply not having that connection with the patient to break through. I think that’s the biggest trigger for me when it comes to feeling CF” (P7). This reinforces feelings of powerlessness and an inability to deliver optimal care, which makes caregiving less satisfying.

#### 3.3.3. Healthcare System Under Pressure

Participants indicate that the healthcare system is under pressure, which is an important contextual factor contributing to CF. This mainly includes high workloads, time constraints, insufficient staffing levels, and administrative burdens. Another contributing factor is the fear of making errors. While midwives express concerns about digitalization, gynecologists express concerns regarding the legal repercussions in the event of a medical error. Both report that due to these perceived demands, they cannot focus adequately on what they perceive as truly meaningful, which is person-centered care: “The core of caregiving has been pushed to the background. And that means people who choose this profession—let’s focus on midwives and gynecologists here- are no longer able to fully engage with the core of that profession. And that exhausts them very much” (P3).

### 3.4. Protective Factors

#### 3.4.1. Supportive Team Environment

The team environment and functioning, were highlighted as key protective factors against CF: “And that’s the moment when I could have experienced it [CF], but then you keep going because you don’t want to let the group down either” (P6). According to participants, team members could be a significant source of support. Many emphasize the need for a safe and open team atmosphere where one can discuss their issues: “The team I was in, we could say things to each other like ‘Yeah, I don’t really like what you’re saying about that woman.’ That was okay. It was really an open atmosphere like that” (P5). Additionally, participants emphasize the importance of shared responsibility within the team and being able to rely on each other: “That you feel like ‘I’m having a hard time keeping my calm’, […] that you just ask colleagues for help to take over for a moment” (P8).

Some participants recognized the presence of a stigma attached to seeking help, in particular psychological help, as taboo. This perception persists even though professionals recognized the importance of seeking help. The majority of participants preferred informal support from colleagues over formal psychological support: “We can also visit the hospital psychologist if we want to […] It does happen, but you also know that, for some reason, there is a little bit of shame surrounding it. So it’s like no one really talks about it “ (P4).

#### 3.4.2. Compassion Satisfaction

Participants derive great satisfaction from the affirmation and gratitude they receive from patients. What matters most to them is the impact they can make to the birth experiences of their patients: “Those women are lying there with the worst pain you can have, and you are trying to resolve it and if everything goes well and the child is well, then you are also a bit of a hero, aren’t you?” (P4). Witnessing the birth of a newborn and being part of the labor and delivery process is seen by many as deeply meaningful: “I can really enjoy a beautiful birth” (P9).

#### 3.4.3. Job Autonomy and Flexibility

Job characteristics may enhance overall job satisfaction. Participants valued autonomy, including flexible or self-arranged work schedules, and ownership (e.g., making decisions, giving input, seeking opportunities for professional growth): “We actually work very independently, which is really nice, because it allows you to do your thing. I’m allowed to think for myself, to suggest ideas. […] I need that kind of autonomy to keep feeling challenged in my job.” (P7).

#### 3.4.4. Personal Coping Skills

Various individual coping strategies were recommended to prevent CF. These involve cultivating optimism, humor and appreciating simple pleasures: “I try to enjoy the little things like, what might be everyday stuff for someone else, for me it’s having a good coffee, for example.” (P6). A solution orientation is also described as a helpful trait. To ensure proper care for patients and colleagues, prioritizing self-care is imperative, according to participants. They acknowledge the significance of listening to their emotions and body to ensure adequate rest: “If you experience that CF, I think it’s also difficult to provide care, because if you’re exhausted yourself, how can you still care for others? So I think it’s really important to take a moment for yourself, and if you’re okay with that, if you feel good about it and are at peace with it… Only then you can care for someone else “ (P6).

#### 3.4.5. Awareness

Participants emphasized raising awareness about CF to reduce stigma and facilitate access to support. In their opinion, midwives and gynecologists should have comprehensive knowledge about the concept of CF and should be able to identify its symptoms: “I’m thinking, for instance: how do you notice within yourself that you are reaching your limits or what are the things you need to pay attention to? And what are you going to do?” (P8). The key skill identified for teams to avoid CF is to communicate in an open and safe team environment. A desire was expressed for communication skills training to better equip them for challenging situations in maternity care. Several participants highlighted the debriefing and constructively providing and receiving feedback: “Opening up the subject, communication, I think. Just bringing it up as well and not covering it up, but also just really talking about it.” (P6).

## 4. Discussion

This study explored the experiences of midwives and gynecologists in Flanders regarding CF, identifying three themes: experiences, risk factors, and protective factors. These results offer insights into challenges faced by maternity care professionals and potential interventions to improve both caregiver well-being and patient care experiences. In line with Figley’s definition of CF [5,6], our findings illustrate how sustained empathic engagement can lead to emotional depletion and diminished capacity for empathy. This supports the notion that CF is rooted in relational exposure to suffering rather than in workload alone, as is the case with general occupational burnout [7,10].

Limited familiarity with the term CF highlights the need for targeted training and awareness. On a personal level, emotional exhaustion and reduced empathy and job satisfaction are experienced. The emphasis on empathy reduction as a core experience of CF underscores the unique nature of CF in caregiving professions, differentiating it from burnout and STS [2,3,4,5,6,7,8,9,10,11]. Reported experiences of CF were characterized by a perceived “inability to provide care as one normally would”. It manifests as more routine and less person-centered care and can impact team functioning through increased absenteeism, negative team atmosphere and a sense of collective exhaustion. This aligns with the literature on the impact of CF on healthcare systems, where it contributes to medical errors and decreased quality of care [4,9].

The perception of certain patients as being ‘energy-consuming’ may function both as an antecedent and an early manifestation of CF. Although the current study design does not allow for causal inference, the literature suggests that this perception may be part of a circular, dynamic process [2,3,4,7,10,26]. Care providers may initially feel deeply connected and responsible, but over time begin to experience patients as overwhelming or draining as their internal coping capacity diminishes. This shift in perception may in turn reinforce the sense of burden and further accelerate the onset of CF, creating a feedback loop. This dynamic is also described by Sinclair et al. (2007) as a cyclical erosion of empathic capacity rather than a linear endpoint [7]. This is particularly likely when contextual stressors, such as pressure on the healthcare system, are present and protective factors, such as team support, are lacking.

Many participants reported experiencing pressure on the healthcare system as a contextual factor, a finding that aligns with literature identifying high occupational use of self and high stress exposure as key antecedents of CF [4]. Notably, the global COVID-19 pandemic placed high emotional and organizational demands on maternity care providers, the effects of which appear to remain tangible even in the aftermath of the acute crisis [27]. In this study, midwives and gynecologists noticed that an increase in workload and administrative demands—particularly electronic medical records—consume valuable time that could otherwise be invested in meaningful patient care. These digital systems, though designed for efficiency, were seen as limiting autonomy and undermining the ability to provide person-centered care, potentially contributing to burnout [28]. Research in the Netherlands also recognizes the amount of administrative and organizational tasks as an important reason for midwives’ intention to leave the job [29]. As one participant in our study suggested, a possible solution could be the provision of administrative support so healthcare professionals can focus on the core tasks of providing care [27]. Additionally, balancing workloads across staff, allowing for autonomy and enabling flexibility, could enhance job satisfaction and prevent CF and burnout [14,28].

Gynecologists reported a fear of making mistakes—intensified by digitalization and monitoring—as a source of stress. They described how this pressure increased hesitation and caution, which paradoxically increased the risk of errors. This aligns with literature suggesting that highly medicalized environments, such as maternity care, can contribute to emotional strain and a reduced sense of professional autonomy [12,30]. Research shows that such contexts may lead to defensive practices, hesitation in decision making, and over-reliance on protocols—all of which can impair critical thinking and clinical judgment [28]. Thus, the fear of making errors may be a consequence of CF as well as a contributing factor to its development. This circular process further highlights the importance of understanding CF as a systemic issue which requires structural changes in how care is organized, including attention to workload, autonomy and a culture of psychological safety.

Midwives and gynecologists emphasized trust and connection within the maternity care team as the most important reported buffer against CF. Collaboration and social support helped alleviate stress arising from challenging birth experiences. This finding aligns with research among nurses demonstrating that team cohesiveness is linked to higher compassion satisfaction and serves as an effective buffer against burnout and CF [31,32,33]. In addition, supportive team dynamics are known to foster resilience by strengthening individuals’ ability to recover from emotionally demanding situations. Consistent with research in midwives, participants preferred peer support from direct colleagues over formal support from external services [34].

A crucial element underlying support is the presence of psychological safety within teams, defined as the shared belief that the team environment is safe for interpersonal risk-taking [35]. High psychological safety allows professionals to speak up, report mistakes and share feelings without fear of blame or ridicule, fostering open communication [36,37]. Such a climate enhances team learning and success and promotes patient safety [38]. Within this context, structured and informal debriefing opportunities after stressful incidents or work-related conflict emerged as key processes enabling professionals to process emotions and reflect on experiences. Debriefing serves individual emotional regulation and strengthens team cohesion, thereby disrupting potential negative feedback loops that exacerbate CF.

Another finding was that midwives and gynecologists viewed their contribution during pregnancy and delivery as inherently meaningful and fulfilling. Witnessing new life and providing emotional support were described as a core source of professional motivation, this experience of meaning is tied to compassion satisfaction (CS)—the pleasure derived from helping others and making a meaningful difference [26]. Such meaning-making buffers against emotional exhaustion, fosters resilience, aids recovery from stress and maintain engagement [39]. Higher levels of CS are linked to reduced turnover and greater retention, especially in emotionally demanding fields like maternity care [28]. Environments that promote both CS and psychological safety may strengthen individual and team resilience, helping to prevent CF and support long-term workforce commitment. This interpretation aligns with results from Sinclair et al. (2007), which position CS as the principal buffer against CF [7].

This study is one of the first to explore CF among maternity care professionals in Flanders. A key strength lies in the qualitative, exploratory design, which enabled an in-depth, nuanced understanding of complex emotional processes that are often underreported in survey-based research. The sample included professionals from various care settings, enhancing the diversity of perspectives captured. Another strength was the use of investigator triangulation during coding and theme development, which contributed to the credibility and confirmability of the analysis. The iterative development and refinement of the codebook ensured a grounded and collaborative approach to theme identification.

Despite these strengths, several limitations must be acknowledged. Results from this study cannot be readily generalized nor considered as reflecting the full array of experiences in the domain of CF in obstetric teams, given the small sample size and selected sample. When transferring these findings, contextual details (e.g., organization of healthcare and staffing models) should be compared and taken into account. Gynecologists, especially males, were underrepresented in this study sample. A notable limitation is the potential for selection bias. While none of the participants were initially familiar with the concept of CF, recruitment may have attracted individuals with a heightened interest in caregiver well-being or workplace stress. Because participants were unfamiliar with CF as a distinct concept, they could not clearly identify personal experiences as such. Overlap with burnout likely shaped how they described their experiences, making it difficult to distinguish CF-specific aspects. Findings should therefore be seen as reflections of emotional strain consistent with, but not unique to, CF. Finally, although the use of Microsoft Word and Excel for data analysis allowed for iterative engagement with the data, these programs are less sophisticated than specialized analysis software. The absence of such software may have limited analytical depth (such as relationships between codes and themes) and functionalities (such as visualization of code relationships), which is acknowledged as a methodological limitation.

## 5. Conclusions

Our study pointed out that CF is an unfamiliar concept but likely to be a problem experienced within maternity care teams, which is perceived to negatively impact patient care. Future research should focus on developing and evaluating strategies for early detection—both self-recognition and recognition by colleagues. Initiatives to prevent CF could be aimed at improving awareness, emotional literacy, compassion satisfaction and communication skills to support timely identification and intervention. Particular attention should be paid to fostering a supportive team environment, as participants consistently described team dynamics as a crucial buffer against emotional strain. Research should further explore how teams can be equipped to respond constructively to signs of distress, and how leadership can cultivate a psychologically safe climate where CF can be discussed openly. Finally, we call for broader organizational and policy-level interventions that address systemic drivers of CF, such as staffing constraints and excessive workload. An integrated approach that combines individual, team-based, and systemic responses is essential to sustainably mitigate CF in maternity care.

## Figures and Tables

**Table 1:** Participant characteristics.

Participant Number	Midwife (M) or Gynecologist (G)	Area of Practice	Years of Professional Experience
1	M	Hospital setting	13
2	M	Community care	27
3	G ^1^	Private practice	30
4	M	Hospital setting	14
5	M	Private practice	15
6	M	Hospital setting	9
7	M	Hospital setting and private practice	6
8	M	Hospital setting	15
9	G	Hospital setting	8
10	G	Hospital setting and private practice	11

^1^ Male participant.

## Data Availability

The raw data supporting the conclusions of this article will be made available by the authors on request.

## Supplementary Materials

The following supporting information can be downloaded at: https://www.mdpi.com/article/10.3390/healthcare13222908/s1, File S1: Topic guide. Reference [25] is cited in the supplementary materials.

## Abbreviations

The following abbreviations are used in this manuscript:

CF	Compassion fatigue
CS	Compassion satisfaction
STS	Secondary traumatic stress
MSc	Master of Science
