# Supplementary material for: Providing Compassionate Care: A Qualitative Study of Compassion Fatigue Among Midwives and Gynecologists
Source: Healthcare (Basel). 2025 Nov 14;13(22):2908. doi: 10.3390/healthcare13222908 (PMC12652111; doi:10.3390/healthcare13222908)
Supplement: Supplementary file 1 [file healthcare-13-02908-s001.zip › healthcare-3887178-supplementary.pdf]

Topic guide

1. Introduction

Thank you very much in advance for participating in this project. In this study, we want to learn more about the prevalence of Compassion Fatigue among gynaecologists and midwives and how to counteract its development.
During this interview, we will ask you some questions about your experiences with compassion fatigue. We intend to use these interviews with birth care professionals to develop a simulation training programme to counter compassion fatigue and strengthen resilience in the workplace.
I will get you started by asking some questions, but so there are certainly no right or wrong answers. Anything you think or feel that is related to the questions for you, you may tell me.
If you prefer not to answer something, or if you want to add to something, or you want to return to something, just say so! If you would like some more explanation, or you want to stop the interview for a while, also be sure to let us know. Feel free to take your time to think, it is not at all bad if there are silences, because those moments are also sometimes important for me so that I can think carefully about the next question.
Everything you tell me is completely confidential and will remain completely anonymous. To make processing the interview easier, I would like to record our conversation, is this OK with you? After the study, all recordings will be deleted.

Were you able to read through the informed consent or would you prefer to go over it again? You are always welcome to contact us if you have any further questions. May I ask you what time at the latest you would like to finish the interview so that we can finish in time? Would you like a summary of the results at the end of the study?
After this, the informed consent form will be signed in duplicate.

Start sound recording

2. Personal experiences with compassion fatigue

- Can you tell us something about your job content? What does your job responsibilities entail?
- When I say compassion fatigue, what do you think of?
- Now I will read out our definition of compassion fatigue for you: ‘Compassion fatigue is a state of physical and mental exhaustion that occurs in caregivers within a caring relationship. Consequences can include a decrease in empathy, emotional exhaustion, sleep disturbances, hyper-vigilance, anxiety, fear, difficulty concentrating, physical complaints such as muscle tension, feeling overloaded or tired, isolation and detachment.’
- Have you ever experienced compassion fatigue?
    - If so, what was your experience? What signs do you notice in yourself?
        - Can you give an example of a situation in which you would experience compassion fatigue?
        - Did your experience of compassion fatigue have an impact on the performance of your work? For example, do you perform some actions or interactions with patients or colleagues differently than you would like to do?

  - If not, can you imagine a situation in which you might experience compassion fatigue?
      - Are you more likely to experience this when: a stressful or emotional situation occurs; when something is asked or expected of you by patients or colleagues (e.g. advice, reassurance, comfort); at the end of your shift or week
    - Can you give an example of a situation where you could have experienced compassion fatigue, but for some reason this did not happen?
    - Why do you think you were able to avoid experiencing compassion fatigue in this situation?

3. Individual coping strategies to deal with compassion fatigue

- Do you have strategies to deal with compassion fatigue?
  - If yes, what are these?
- Do you have any advice for other birth care professionals on how to deal with compassion fatigue?

4. Factors in work environment that help or hinder in relation to compassion fatigue

- Is compassion fatigue a concept discussed at your workplace?
  - If discussed, how is it addressed?
- What factors at your workplace help you deal with compassion fatigue?
- What reactions would you get from your colleagues or your boss if you told them you were experiencing compassion fatigue?
- Have you already experienced difficulties in letting go of thoughts about work in your private time?
  - What makes it difficult to let go of these thoughts?
  - What makes it easier to let go of these thoughts?
- Do you think compassion fatigue is common in your job?
  - If yes, why is it so?
  - If no, why not?
- What do you find rewarding in your job? What do you get satisfaction from?
- What do you find tiring in your job?
- How sustainable do you consider your current job situation? Can you continue in this job for a long time?
  - What do you think could make it more sustainable?
  - What do you think would make it less sustainable?

5. Impact compassion fatigue on the team

- Do you think compassion fatigue has an impact on your team collaboration?
- Do you think you would recognise compassion fatigue in a colleague?
  - How do you think these symptoms would manifest themselves?

6. Impact compassion fatigue on personal life

- Do you think compassion fatigue has affected your personal life?

    - If yes, in what way?
- Have you ever experienced hopelessness when trying to help your patients
    - If so, in what way did this show itself?

7. Training

Based on these interviews, we want to develop a team training that will be tested in two partner hospitals. What components do you think a training to prevent compassion fatigue and support resilience should definitely include?

8. Closing

I've come around to my questions, are there any issues that have not yet been addressed?
Thank you for your valuable participation!

Stop recording

Reference

1. Harling, M.N.; Högman, E.; Schad, E. Breaking the taboo: Eight Swedish clinical psychologists' experiences of compassion fatigue. *Int. J. Qual. Stud. Health Well-being* **2020**, *15*(1), 1785610. https://doi.org/10.1080/17482631.2020.1785610
